# Effect of Hepatitis E Virus on the Male Reproductive System: A Review of Current Evidence

**DOI:** 10.3390/v17010066

**Published:** 2025-01-05

**Authors:** Ahmed A. Kotb, Mohamed A. El-Mokhtar, Ibrahim M. Sayed

**Affiliations:** 1 Department of Microbiology and Immunology, Faculty of Pharmacy, Assiut University, Assiut 71515, Egypt; ahmedabedelaziz994@aun.edu.eg; 2 Gilbert & Rose-Marie Chagoury School of Medicine, Lebanese American University, Byblos P.O. Box 36, Lebanon; 3 Department of Biomedical & Nutritional Sciences, Zuckerberg College of Health Sciences, University of Massachusetts Lowell, Lowell, MA 01854, USA

**Keywords:** Hepatitis E virus, extrahepatic manifestations, male reproductive system, semen, male infertility, testicular tissues

## Abstract

Hepatitis E Virus (HEV) is a globally widespread pathogen that causes acute hepatitis infection. Beyond hepatic pathogenesis, HEV has been proven to cause several extrahepatic manifestations, such as neurological, renal, and hematological manifestations. It was also associated with mortality in pregnant females. Several studies have investigated the impact of HEV on the male reproductive system; however, the available data are limited and conflicting. Assessment of the patients’ ejaculates/semen samples revealed that HEV particles are excreted in these fluids in cases of chronic infection but not acute infection. The excreted HEV particles are infectious to in vivo animal models and in vitro cell culture. However, the effect of HEV infection on male infertility is not confirmed. One study including human samples showed male infertility associated with HEV genotype 4 infection. Studies of HEV infection in animal models such as pigs, gerbils, and mice showed that HEV infection caused distortion on the testes, damage of the blood–testis barrier, and induction of inflammatory responses leading to abnormalities in the sperm. The excretion of HEV in the semen fluids raises concerns about HEV transmission via sexual transmission. However, all available data do not confirm the transmission of HEV through sexual intercourse. This review aims to summarize and critically assess the available studies investigating the influence of different HEV genotypes on the male reproductive system, providing insights into whether HEV contributes to reproductive impairment in men.

## 1. Introduction

Hepatitis E Virus (HEV) is an emerging pathogen that is considered the most common cause of acute viral hepatitis worldwide [1,2,3]. HEV, a positive sense single-strand RNA virus that infects humans and animals, is a member of the genus *Paslahepevirus*, subfamily *Orthohepevirinae*, and family *Herpesviridae* [4,5]. The genus *Paslahepevirus* includes two species: *Paslahepevirus alci* and *Paslahepevirus balayani. Paslahepevirus balayani* includes eight genotypes, and five genotypes can infect humans [4,5]. HEV genotypes 1 and 2 are fecal-orally transmitted viruses associated with outbreaks in developing countries [1,6]. HEV genotypes 3, 4, and 7 are zoonotic viruses, and pigs, wild boars, camels, and rabbits are the main reservoirs and sources of human infection, mainly through foodborne transmission [7,8,9,10,11]. Blood transfusion can be another source of HEV infection in humans [12,13,14,15]. Vertical transmission is confirmed from pregnant women to their fetuses, causing severe complications in the case of HEV genotype 1 infection [16,17] and mild infection in the case of HEV genotype 3 infection [18,19]. The genome of HEV contains three open reading frames, and the genome of HEV genotype 1 includes four open reading frames [20,21]. Open reading frame 1 (ORF1) is located at 5’ of the genome, and it encodes the proteins required for viral replication (non-structural proteins) such as RNA polymerase, helicase, and cysteine protease [21]. Open reading frame 2 (ORF2) is located at 3’ of the viral genome and includes the structural proteins required for capsid and virus assembly [22,23]. There are three different forms of ORF2 proteins; two forms are associated with non-infectious virions, and they are glycoprotein (ORF2g and ORF2c), and one form is associated with infectious virions and not linked with glycoprotein (ORF2i) [24,25,26]. Open reading frame 3 is required for viral infection, replication, and release [27]. Open reading frame 4 stimulates the RNA polymerase and activates the viral replication [20].

HEV can cause acute and chronic infections. Acute HEV infection, mainly caused by HEV genotypes 1 and 2, is mostly a self-limiting disease; however, progression to acute liver failure can develop, especially among pregnant women, as well as coinfection with other hepatotropic viruses and old ages [16,28,29]. It is worth noting that HEV genotypes 1, 2, and 4 can cause acute liver failure in pregnant women, while HEV genotype 3 causes mild infection in pregnant women [17,18,30,31]. HEV can persist in the human body for more than three months, leading to chronic infection [32,33,34]. Chronic infection, caused by HEV genotypes 3, 4, and 7, is mainly developed in patients with weak immunity such as patients receiving immunosuppressant drugs after organ transplantation, patients infected with HIV, and patients with leukemia [32,33,34]. HEV genotypes 1 and 2 do not cause chronic infection [35,36]. Chronically HEV-infected patients are at risk of developing extrahepatic complications, mainly in the nervous system, kidney, placenta, bone marrow, and monocytes [37,38,39,40,41,42]. Neurological manifestations can be caused by direct replication of the virus (such as meningitis or encephalitis), immune responses against the virus such as Chronic Inflammatory Demyelinating Polyneuropathy (CIDP), or the cause of some manifestations (such as neuralgic amyotrophy and Guillain–Barré syndrome) is not clear due to the viral replication or host immune responses [42]. Additionally, the genital system is also a target for HEV replication. HEV genotypes 1, 3, and 4 have been shown in earlier research to have an impact on the reproductive system of females. In vivo animal studies demonstrate the ability of HEV genotype 4 to multiply in the ovaries and uterus, which facilitates the vertical transmission of HEV to the fetus [43,44]. HEV genotype 1 and, to a lesser extent, genotype 3 have been shown to replicate in decidual and fetal placental stromal cells as well as in organ cultures, causing significant tissue damage and altering the cytokines secreted in the surrounding microenvironment [45]. Moreover, HEV genotypes 1 and 3 can replicate efficiently in primary human endometrial stromal cells, suggesting that these cells could be an endogenous source of the infection during pregnancy [46]. The extrahepatic disorders associated with HEV infection are either mediated by direct pathogenesis and replication of the virus or indirectly through the formation of virus–antibody immunocomplexes that can precipitate in various organs and stimulate inflammatory responses [37]. Regarding the effect of HEV on the male reproductive system, the role of HEV in male infertility is a query that has not been answered. The data about this hot topic are scarce and not conclusive, probably due to the difference in the viral genotypes, experimental approaches used, source of the virus, etc.

Male reproductive health can be significantly impacted by viral infections, which may result in infertility and other complications. Various viruses have been shown to adversely impact men’s reproductive health, hormonal balance, and sperm quality [47]. Several viruses, including the Hepatitis B virus, Hepatitis C virus, Human immunodeficiency virus, Human papillomavirus, Human simplex virus, Human cytomegalovirus, Zika, Mumps virus, influenza, Ebola, and SARS-CoV-2 virus, have been linked to infection-induced male infertility [47,48]

**Figure 1:**
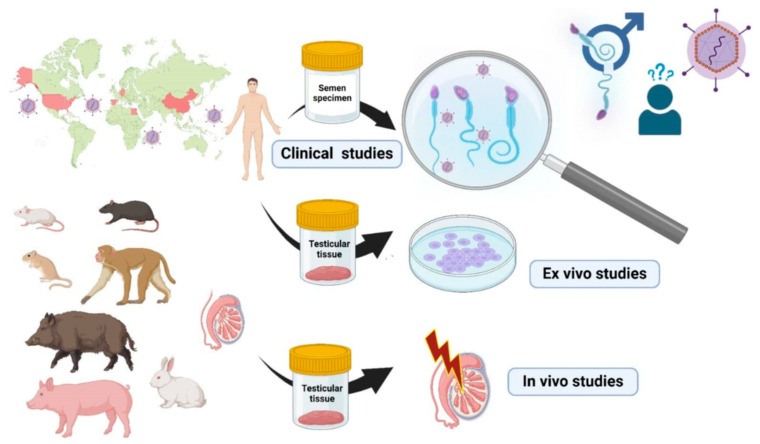
Experimental approaches used for studying the effect of HEV infection on the male genital system.

In this review, we will discuss the effect of HEV infections on the male reproductive system and the excretion of HEV in the semen samples, which raise concerns about the possibility of HEV transmission via sexual intercourse. We collected articles that assessed HEV in patients’ samples and in vivo and in vitro model systems used to evaluate the possibility of HEV infection in the male reproductive system as well as the infectivity of HEV particles excreted in these organs or fluids (summarized in Figure 1).

## 2. Effect of HEV Infection on the Male Reproductive System and Excretion in Semen

### 2.1. Studies That Include Patients’ Samples

A small number of studies conducted in various countries have examined the prevalence of HEV in semen samples and the effect of infection on the male reproductive system yielding contrasting results. However, it is not clear if this contraindication was due to the patient cohort, tested patients, virus genotype, stage of diseases, etc. These findings are discussed further in this section of the review and summarized in Table 1.

#### 2.1.1. Studies Report an Effect of HEV Infection on the Male Reproductive System and Excretion in Semen

In 2018, a study was conducted in Kunming, China, which analyzed semen samples of 185 infertile men and found a high prevalence of HEV RNA (28.1%) [54]. The viral titers in semen were comparable to those detected in urine. Phylogenetic analysis showed that it was of genotype 4 h. This isolate was highly similar to the HEV isolate circulating among pigs and ruminants in the same region [54]. Moreover, a higher HEV seroprevalence was recorded in infertile men than in the general population and pregnant women in this region [54]. Importantly, the author highlighted that HEV infection impaired semen quality, decreased motility and vitality, and increased the percentage of abnormal sperm [54]. Computer-assisted semen analysis (CASA) revealed that 97% of the sperm of HEV-infected patients were asthenospermia [54] and about 54% of the oligospermia patients were HEV RNA-positive [54]. However, future studies need to confirm these findings. Another study was performed in Germany; the authors analyzed the blood, urine, stool, and ejaculate from chronic HEV-infected patients (*n* = 3) and acute HEV-infected immunocompetent patients (*n* = 6) [52]. HEV RNA was detected in the ejaculation of two out of three chronically infected individuals in both semen and seminal plasma but not in the acute immunocompetent patients [52]. Later, the same group assessed another small cohort of immunocompromised patients (*n* = 6), and five out of the six patients also tested positive for HEV RNA in the patient ejaculation [51]. The isolated HEV subtypes belonged to 3c, and one isolate was not classified and assigned to 3abjk, but the isolates from the patients who tested negative for HEV in the ejaculate were 3i (*n* = 1) and 3c (*n* = 1) [51,52]. The HEV loads were higher in seminal plasma and semen (2–3 logs) than in urine [52]; the load reached 10^7^ IU/mL in the ejaculate of three patients and the blood of one patient [51,52]. The HEV titer was 100 times higher in the ejaculate than in the serum in five patients, while the titer was lower in the ejaculate than in the serum in two patients [51]. Electron microscopy examination and density gradient approaches revealed that the HEV particles excreted in the semen were enveloped, similar to the particles excreted in the blood (enveloped) and different from the naked non-enveloped released in the patients’ feces [52]. However, by sequencing and multiple sequence alignment, the HEV particles excreted in the ejaculates were genetically different by 15 amino acids than the particles in blood or stool, mainly in the hypervariable region of ORF1 [52]. The viral particles excreted in the ejaculate did not show infectivity to HepG2/C3A cells in vitro [52] but were infectious to optimized PLC/PRF/5 cells [51]. However, HEV RNA was not detected in the semen of immunocompetent HEV-infected patients [52]. 

Interestingly, after ribavirin therapy, HEV could be detected in the ejaculate of one patient even after the clearance of the virus from blood and stool [52]. HEV RNA was detectable in the ejaculation of this patient after stopping ribavirin [52]. The previous findings suggest that HEV could persist in the semen for a longer period than the blood and stool, and the ribavirin could differentially affect HEV loads in different compartments. Since the findings of this study were derived from a small cohort, further studies should confirm these data.

#### 2.1.2. Studies Report No Effect of HEV Infection on Male Reproductive System and Male Infertility

Horvatits and colleagues assessed HEV RNA in the semen of infertile men (*n* = 87; 79 retrospective, and 8 samples were prospective), and the authors did not detect HEV RNA in any of these samples, concluding that there is no link between male infertility and HEV genotype 3, which is the common genotype circulating in Europe [53]. Likewise, Wang and colleagues prospectively analyzed the semen of infertile men (*n* = 1183), collected from the Department of Reproductive Medicine Centre, Peking, China (2018–2019), for the presence of HEV RNA. None of the tested patients were positive for HEV [55]. Since HEV genotype 4 is the common genotype in China [13], the authors concluded that the link between HEV genotype 4 and male infertility is weak [55]. In a parallel line, EL. Mokhtar et al. retrospectively analyzed the semen and blood of infertile patients (*n* = 120) for HEV markers including HEV RNA, HEV Ag, and HEV-antibodies and these patients were negative for these markers [49]. Moreover, the same study prospectively assessed HEV markers in the blood, urine, and semen of acute HEV-infected patients (*n* = 25), and there was no evidence of HEV markers in the semen of acute HEV-infected patients, though some makers were positive in the blood and urine of the same patients [49]. In addition, there was no difference in the level of reproductive hormones including follicle-stimulating hormones (FSH), luteinizing hormone (LH), testosterone, prolactin, and estradiol between the HEV-infected patients and healthy control subjects. Moreover, sperm quality was not impacted by HEV infection, and sperm motility, abnormal form, and liquefaction time were comparable in both groups [49]. Collectively, these authors concluded that HEV genotype 1 (the most prevalent genotype in Egypt [29,56]) is not harmful to the male reproductive system [49].

### 2.2. Ex Vivo and In Vitro Studies

Researchers used human hepatoma cell lines to assess the infectivity of HEV particles in the patients’ ejaculation. Also, testes explants and primary human Sertoli cells were used to evaluate the infectivity of HEV in these organs, summarized in Table 1.

#### 2.2.1. Human Hepatoma Cell Lines

Hepatoma cell lines such as HepG2/C3A, PLC/PRF/5, and Huh7.5 are the most common hepatoma cell lines that support HEV replication [25,26,57]. These cell lines are permissive to stool-derived HEV preparations, plasma-derived HEV preparations, and HEV cell culture preparations [26,58]. Hepatoma cell lines were used to assess the infectivity of HEV particles excreted in the semen. Horvatits et al. used the HepG2/C3A cell line to assess the infectivity of HEV particles in the ejaculates of two immunocompromised patients; one patient underwent a heart transplant, and the second patient had chronic lymphatic leukemia [52]. Both patients were chronically infected with HEV genotype 3 subtype c. HepG2/C3A cells were not permissive to the ejaculation of the previous patients [52]. Also, stool, plasma, and urine from those two patients were not infectious to the cells [52]. Later, Schemmerer et al. used an optimized cell culture system to assess the infectivity of HEV particles in the patients’ ejaculates. This optimization includes the use of overconfluent PLC/PRF/5 cells, to which supplements were added such as amphotericin B, fetal calf serum, and distinct salt to enhance the infectivity and virus production [57]. Schemmerer et al. retested the infectivity of two patients’ samples that were not infectious to HepG2/C3A cells [51,52]. The ejaculate of the first patient (heart transplant [52]) was infectious to PLC/PRF/5 cells as the HEV RNA was increased in the culture supernatant at day 14 post-infection and the titer increased till day 70 post-infection [51]. Moreover, the extracellular HEV ORF2 antigen was detected in the culture supernatant and the level was increased with time, indicating robust infection [51]. In addition, HEV ORF2 was detected intracellularly in the infected cells by immunofluorescence [51]. Importantly, the ejaculate supernatant and ejaculate lysate are infectious to PLC/PRF/5 cells; however, the ejaculate lysate was more infectious than the ejaculate supernatant, probably due to the particles in the former one were naked particles [51]. The ejaculate of the second patient (chronic lymphatic leukemia [52]) was not infectious to overconfluent PLC/PRF/5 cells [51]. Then, Schemmerer et al. assessed the infectivity of the ejaculate of other HEV-infected patients (*n* = 5) using this optimized culture media, and they were infectious to the cells in vitro [51]. However, the infectivity was varied based on patients’ samples [51]. In one patient (kidney transplant, infected with HEV genotype 3 subtype c), the patient’s fecal preparation was less infectious than the ejaculate and the urine from this patient was not infectious to PLC/PRF/5 cells [51]. In another patient (Lymphoma, HEV genotype 3, unassigned subtype), the patient’s fecal preparation was more infectious than the ejaculate to PLC/PRF/5 cells [51]. The RNA load in the PLC/PRF/5 culture supernatant reached 10^9^ copies/mL in the supernatant of cells challenged with the patient’s ejaculate, suggesting the infectivity and replicative competence of HEV from the ejaculate [51]. The difference between the infectivity of HEV particles among patients’ materials could be attributed to the difference in properties of HEV particles (naked, enveloped), viral load, patient’s medication history, storage condition, etc.

#### 2.2.2. Human Testicular Tissues (Testis Explants)

Li and colleagues studied the susceptibility of stool-derived HEV genotype 3 (subtype 3b), stool-derived HEV genotype 1 (subtype 1b), and stool-derived rat HEV (HEV-C1) to testis explants isolated from healthy donors [50]. HEV genotype 3 replicates efficiently in 70% (7 out of 10 donors) of the testis explants, as indicated by the detection of HEV RNA in the culture supernatant day 1 post-infection and detection of HEV ORF2 intracellularly [50]. HEV ORF2 was detected mainly in the germ cells, Sertoli cells, and seminiferous tubules [50]. Interestingly, treatment of testis explants with immunosuppressant (tacrolimus) increased the infectivity and permissiveness of the testes to HEV genotype 3; 100% infection (10 out of 10 of donors) was attained and the viral load was significantly higher after 1–3 days in treated cells than non-treated cells [50]. The viral particles released in the culture supernatant of testis explants were infectious as they could establish the infection in gerbils [50]. In contrast, testis explants were not permissive to stool-derived HEV genotype 1 and rat HEV [50]. Finally, HEV genotype 3 infection induced damage in the testis explants, distorted germ cells, and slightly stimulated pro-inflammatory responses, except IL-18, which was highly up-regulated [50].

#### 2.2.3. Primary Human Sertoli Cells

HEV genotype 3 can replicate efficiently on primary human Sertoli cells, and the RNA titer increased in the culture supernatant [50]. In contrast, HEV genotype 1 cannot replicate in these cells [50]. The addition of tacrolimus enhanced the replication of HEV genotype 1 and genotype 3. Efficient replication of HEV genotype 1 was only successful after tacrolimus treatment [50]. The efficiency of replication was higher in HEV genotype 3 compared to HEV genotype 1 as the viral load was higher in the former one with time [50]. Unlike testis explants, HEV genotype 3 infection induced significant changes in the secretome of Sertoli cells, especially in the proinflammatory cytokines, chemokines, and colony-stimulating factors [50]. It is worth noting that the changes in Sertoli’s cytokine microenvironment were achieved without tacrolimus [50].

### 2.3. In Vivo Animal Model Studies on the Effect of HEV Infection on Male Reproductive System

Various animal models have been employed to investigate the effects of HEV infection on different aspects of the male reproductive system.

#### 2.3.1. Pigs

Pigs are the main reservoir for HEV genotypes 3 and 4 [59,60,61]. Previous studies showed that HEV can replicate in the extrahepatic tissues in pigs such as the intestine, kidney, spleen, and lymph nodes [62]. Li and colleagues were the first study to assess HEV in pig semen (*n* = 26) in Shaanxi province, China [63]. In total, 1 sample out of 26 of the semen samples collected was positive for HEV RNA [63]. Phylogenetic analysis revealed that this isolate belonged to genotype 4 subtype 4i, the prevalent sub-genotype reported in the feces and bile of the pigs included in that study [63].

In another study by Yadav et al., HEV was detected in the epididymis and semen of pigs experimentally infected with HEV genotype 3 (US-2 strain) [64]. HEV ORF2 protein was detected in the acrosomal region of the sperm head [64], and the sperms were infectious to hepatoma cell lines in vitro [64]. HEV RNA titers were significantly higher in sperm cell suspensions compared to seminal fluid and bile from the infected pigs [64]. Moreover, analysis of the semen from infected pigs showed that HEV infection was associated with decreased sperm motility, increased abnormal form, and sperm immobility [64]. The authors hypothesized that HEV could infect spermatogonia, impact Leydig and germ cells, damage the blood–testis barrier, and induce inflammation [64].

On the other hand, Horvatits et al. examined HEV RNA in the testes of HEV-infected male pigs (*n* = 12). The pigs were experimentally infected with HEV genotype 3 derived from the liver of naturally infected wild boars in Germany, at different doses of 10^−4^ to 10^−8^ dilutions (equal 2.6 × 10^4^ IU/dose-2.4 IU/dose, respectively) [52,65]. The infected pigs with doses (10^−4^ to 10^−7^ dilutions, *n* = 8) developed viremia (8/8, 100%) and viral excretion in stool and bile (8/8, 100%) [52,65]. Also, HEV RNA was detected in the liver of infected pigs (8/8, 100%), gall bladders (6/8, 75%), spleen (5/8, 62.5%), hepatic lymph node (6/8, 75%), and mesenteric and other lymph nodes [52,65]. However, HEV RNA was not detected in the testis of any of these pigs (0/8, 0%) [52]. In the other four pigs infected with 10^−8^ dilution of the viral inoculum, HEV RNA was not detectable in any organs [52,65]. The authors hypothesized that HEV was not excreted in the semen of experimental infected immunocompetent pigs [52].

The discrepancy between the two studies, especially those including the HEV genotype (genotype 3) as inoculums, is not clear. One possible explanation is the origin of the virus used. Yadav et al. used the gastrointestinal-derived HEV US2 strain, which was isolated from acute hepatitis patients in the US [66,67], while the virus used in the study conducted by Horvatits et al. was derived from the liver of naturally infected wild boars in Germany [65]. Also, the inoculum doses are different in both studies, and the ages of pigs at the time of inoculation were not the same. Moreover, the time of collection of testes tissues was different. Yadav et al. collected tissues at day 84 post-inoculation, while in the other study, the collection of tissues was earlier than this time. Further studies should be conducted to assess the factors that contribute to infection with the possible effect of HEV on the male productive organs in the pig model.

#### 2.3.2. Mongolian Gerbils

Mongolian gerbils are used for studying the pathogenesis of HEV genotypes 1, 3, and 4 [68,69,70,71]. Mongolian gerbils were also used for studying extrahepatic disorders associated with HEV infection such as neurological manifestations [72]. Soomro et al. studied the impact of HEV infection on the structure and function of testes in the Mongolian gerbils’ model. The authors used liver homogenates of swine-derived HEV genotype 4 as inoculums [73]. HEV was detected in the sera of infected animals starting from day 7 post-inoculation till day 42, while HEV was recorded in the testes of infected animals one week later (day 14-day 42 post-inoculation) [73]. Negative strands of HEV RNA, HEV ORF2 antigen, and HEV ORF3 antigens were detected in the infected testes, suggesting replication of HEV in these organs [73]. The peak of HEV RNA titers in the testes ranged from 4.65 at day 28 post-inoculation to 6.23 at day 42 post-inoculation [73]. The serum level of testosterone was significantly lower in the infected animal compared to non-infected control animals on day 21- 42 post-inoculation [73], while the level of estradiol was comparable in both groups in all time points (day 7-day 56 post-inoculation) [73]. The diameter of seminiferous tubules and the level of abnormal sperms were notably higher in the HEV-infected animals [73]. There were clear pathological abnormalities in the testes of HEV-infected animals such as necrosis in the spermatogonia, shedding of germ cells, development of multinucleated giant cells, programmed cell death, etc. [73]. Electron microscopy examination for the infected testes revealed severe DNA fragmentation in the cells with the formation of apoptotic bodies and increased vacuolation [73]. Moreover, upregulation of apoptosis-related genes such as Fas, FasL, caspase-3, B-cell lymphoma 2 (Bcl-2), and Bcl-2-associated protein X (Bax) was detected in the testis of HEV-infected animals [73]. Collectively, the swine-derived HEV genotype 4 could damage the blood–testis barrier in Mongolian gerbils, causing testicular injury, distortion, necrosis, and apoptosis of several sperm cells [73].

Liu and colleagues also studied if HEV genotype 3 can affect the testes. Three gerbils were challenged with fecal preparations of human-derived HEV genotype 3 subtype b (dose 8 × 10^6^ copies/animal), and the viral load was quantified in the liver, testes, and epididymis three weeks post-infection [50]. HEV RNA was detected in the liver (3/3, 100%), epididymis (3/3, 100%), and testes (2/3, 66.7%) of the infected animals [50]. The viral load was higher in the liver than in the epididymis and testes [50].

Also, gerbils were used to test if the ex vivo human testes explants can produce infectious viral particles following the HEV challenge. To this end, human testes explants (treated with tacrolimus) were challenged with fecal preparation of human-derived HEV genotype 3, and the supernatants were collected after 24 h. and used as inoculum for the animals. Gerbils challenged with the culture supernatants became HEV seropositive after 9–10 weeks (66 days post-inoculation) and HEV RNA was detected in the stool after about 5 weeks (34 days post-inoculation) [50].

#### 2.3.3. Non-Human Primates (Rhesus Monkeys)

Non-human primates are the ideal model for studying HEV isolates that cause infection in humans [74]. Huang et al. used rhesus macaques to study the impact of HEV infection on the male reproductive system and male infertility [54]. Two rhesus macaques were challenged with swine-derived HEV genotype 4. HEV markers (RNA and antigens) were detected in the testes and epididymis of the infected animals, suggesting the ability of HEV to replicate in these sites [54]. Histological examination of the HEV-infected testes and epididymis revealed a loss of the seminiferous epithelium, spermatocytes, and Sertoli cells with increased infiltration of inflammatory cells leading to testis congestion [54]. Additionally, the infection disrupted the transcription factor Ets-variant gene 5 (ETV5), which mediates the function of the blood–testis barrier [75], suggesting damage in the blood–testis barrier [54,75]. Moreover, HEV infection caused a significant drop in serum testosterone levels, which is indicative of functional testicular impairment [54]. The HEV infection-induced decrease in Leydig cell counts could be a factor in lower testosterone synthesis, consistent with the dysregulated testosterone levels observed in infertile men [54].

#### 2.3.4. Balb/c Mice

Some studies report that Balb/c mice cannot support the replication of HEV genotypes isolated from Europe [24,76,77,78]. On the other hand, one group reported that Balb/c is an ideal model for studying the HEV genotype 4, which is isolated from China, and the viral pathogenesis in the male reproductive system, pregnancy, and kidney [43,79,80,81]. Situ and colleagues infected Balb/c mice with a fecal preparation of human-derived HEV genotype 4 and assessed the effect of HEV infection on the male reproductive system [81]. HEV RNA was recorded in the testes after 1 week of infection and reached a maximum after 3–4 weeks of infection and was detectable in the testes longer than in the blood [81]. HEV RNA was also detected in the seminal vesicles, epididymides, and epididymal fluid [81]. The kinetics of RNA in the epididymal fluid was slower (delayed) compared to the testes, i.e., RNA appeared 1 week later in the epididymal fluid. However, the HEV load persisted longer in the epididymides and the epididymal fluid than in sera, feces, and liver (up to day 70–90 post-infection) [81]. The previous finding was probably due to a weak innate immune response stimulated by the virus in the testes compared to the blood due to the immune-privileged nature of the testes [81]. RIG-I, IFN-β, TNF-α, IL-6, and IL-10 were not activated in the testes of infected mice, while IFN-λ was activated [81]. Not only the viral RNA but also HEV ORF2 antigen was observed in the testes, seminal vesicles, and epididymides of HEV-infected mice, suggesting that these sites are permissive to HEV replication [81]. More specifically, the authors reported that testicular peritubular–myoid cells and Leydig cells support HEV replication [81]. Moreover, HEV infection caused a reduction in sperm quality, damage to the blood–testis barrier, spermatogonia decline, and hormonal changes as shown by a reduction in the level of testosterone and inhibin B, suggesting that HEV infection causes serious damage to the testis and epididymal [81]. Importantly, the level of these hormones returned to normal after the viral clearance [81]. Also, HEV-mediated male infertility can be partially reversed after the viral recovery [81].

#### 2.3.5. Rabbits

Rabbits are an ideal model for studying the pathogenesis of rabbit-derived HEV strains, HEV genotype 4, and some isolates of HEV genotype 3 [82,83,84]. Rabbits infected with HEV can develop chronic infection and extrahepatic manifestations in the intestine, spleen, and kidney [85,86]. Rabbits were used as a model for testing the effect of HEV infection on the female reproductive system [44]. Liu and colleagues studied the effect of HEV infection on the intratesticular transcriptome changes in the rabbit model [50]. Chronic HEV infection was successfully developed in immunocompromised rabbits (treated with tacrolimus) and challenged with a fecal preparation of rabbit HEV strain (genotype 3, HEV-3ra) [50]. HEV RNA was detected in the testes of the rabbits at week 13 post-infection. RNA sequencing analysis of the testes collected from HEV-infected immunocompromised rabbits showed downregulation of 887 genes including genes related to spermatogenesis, spermatid differentiation, sperm motility, reproduction, gamete generation, germ cell development, etc. [50].

#### 2.3.6. Other Naturally Infected Animals

Qian and colleagues investigated the presence of HEV genotype C1 (*Rocahepevirus ratti*, rat HEV) in the testes of naturally infected male wild rats, present in Yunnan Province, China [87]. HEV-C1 RNA and antigens were detected in 8 out of 37 (21.6%) testis samples from the wild male rats [87]. Another study by Risalde et al. tested the presence of HEV in the testes of naturally infected wild boars [88]. Wild boars are reservoirs of HEV genotypes 3 and 4 in many countries, especially European countries [89]. HEV RNA and antigens were tested in the blood, testes, liver, and lymph nodes of male wild boar. HEV RNA was detectable in the serum of four animals, with three of these confirmed to be infected with HEV genotype 3f through phylogenetic analysis [88]. Only one animal exhibited detectable viral load in its testis, where HEV-specific labeling was observed in a small number of fibroblasts and some Sertoli cells. Importantly, despite the presence of HEV in the testis, the study did not find any tissue damage associated with the infection [88].

The in vivo animal models used to assess HEV infection on the male genital system are summarized in Table 2.

## 3. Is HEV a Sexually Transmitted Pathogen?

Men having sex with men (MSM) is a high-risk factor for the spread of fecal–oral infections, such as the Hepatitis A Virus (HAV), especially via the analingus route [90]. Since HAV and HEV are excreted in the feces, the transmission of HEV via the sexual route or among MSM with anal practice is therefore questionable. Moreover, the excretion of HEV in patients’ ejaculate raises concerns about the possibility of HEV transmission via sexual intercourse, specifically when the excreted particles are infectious [50,51]. It is worth noting that the infectivity of HEV particles in the ejaculate was comparable to or higher than the infectivity of HEV particles excreted in the patients’ stool and higher than the infectivity of HEV particles circulating in the blood or urine [50,51,52]. Blood transfusion is confirmed as a method of HEV transmission [12,91,92]. Blood-derived HEV particles were infectious to the human liver chimeric mouse model [58]. Also, urine-derived HEV particles were also infectious to animal models such as non-human primates [93]. Therefore, the initial expectation is the possibility of HEV transmission through sexual intercourse. However, previous studies showed that the transmission of HEV through sexual transmission is rare [94,95]. Rodríguez-Tajes and colleagues assessed HEV infection among MSM during acute HAV outbreaks in Spain [94]. HEV markers were tested among MSM (*n* = 83), and there was no difference in the prevalence of anti-HEV IgG between MSM and non-MSM. Moreover, all the samples tested negative for HEV RNA, and only two samples were anti-HEV IgM positive [94]. The authors concluded that the incidence of HEV transmission among MSM is very low (zero incidence) [94]. In Italy, Spada and colleagues assessed HEV markers among MSM (*n* = 196) during HAV outbreaks and blood donors (*n* = 3912) from the same geographic regions [96]. The seroprevalence of HEV was higher among blood donors than MSM, suggesting that sexual intercourse does not play a role in the transmission of HEV [96]. In France, Migueres and colleagues assessed the risk of HEV transmission among MSM who received HIV preexposure prophylaxis (*n* = 147) and blood donors (*n* = 147) of matched age, gender, and area [97]. There was no difference in the seroprevalence of HEV among both groups, and there was no evidence of HEV infection among the MSM, despite the detection of other sexual pathogens including HAV and bacterial infections [97]. Another study was conducted in France on MSM who used HIV preexposure prophylaxis, concluding that HEV infection is not commonly transmitted by sexual intercourse [95]. These findings suggest that sexual transmission of HEV is rare. One possible explanation is that the excretion of HEV in the ejaculate is high among immunocompromised patients, not immunocompetent ones [52]. Only organ transplants and patients with leukemia had positive HEV in the ejaculate [51,52], and these groups were probably not included in the tested MSM groups or the tested MSM groups had not developed chronic infection. Future studies should ascertain these points.

## 4. Conclusions and Future Perspectives

The excretion of HEV in the semen and its role in male infertility and sexual transmission is a hot research topic. Studies on patients’ samples revealed that the possibility of HEV excretion in the semen is high with chronicity, especially in organ transplants and patients with leukemia. However, all the published data include only a few cases. It is important to assess HEV markers in the ejaculate of a larger cohort. To date, the risk of HEV excretion in the semen of HIV-infected patients, another category of immunocompromised patients with a risk of chronic infection, is not known. The HEV particles on patients’ ejaculate seem to be infectious to in vitro optimized cell culture models and animal models. The role of HEV in male infertility has been described in animal models including testicular damage, inflammation, blood–testis barrier breakdown, etc. However, these data have not been confirmed in human samples. Still, the factors contributing to the excretion of HEV particles in the semen are not completely known. For example

(a) Do the viral genotypes and subtypes affect the rate of HEV excretion in the semen?
(b) What is the minimum infectious dose of HEV in the ejaculate?
(c) What are the levels of male sex hormones in chronically HEV-infected patients such as organ transplants and leukemia patients?
(d) Is there any observed deformity in the semen of chronically HEV-infected patients?

Also, it is not known if sexual intercourse could be a natural source of infection, even if the semen is HEV-RNA positive. We believe that this topic needs a lot of investigation and research. Furthermore, understanding the molecular mechanisms underlying HEV’s impact on the male reproductive system is crucial for developing targeted therapeutic strategies.

## Figures and Tables

**Table 1:** Summary of the effect of HEV infection on patients’ male reproductive system and evaluation of the viral infectivity using in vitro cell culture model.

HEV Genotype	Patient Samples		In Vitro Cell Culture	Reference
	HEV in Ejaculate/Semen/Seminal Fluid	Effect on Testis (Male Infertility		
Genotype 1	No	○ No link to male infertility ○ Not affect sex hormones ○ Not affect the sperm quality	○ No replication on human testes explants ○ Replicate in primary human Sertoli cells only in presence of immunosuppressant drugs (tacrolimus)	[49,50]
Genotype 3	○ Yes, in chronically HEV-infected patients (7/9) ○ No, in acutely HEV-infected patients ○ No in infertile men	○ Not recorded, up to our knowledge	○ Replicate in optimized PLC/PRF/5 but not HepG2/C3 ○ Replicate in testis explants, and replication was increased in presence of immunosuppressant drugs. ○ Replicate in primary human Sertoli cells, and replication increased in the presence of tacrolimus	[50,51,52,53]
Genotype 4	○ Yes, in semen of infertile men ○ No, in semen of infertile men	Yes No	○ Not recorded, up to our knowledge	[54,55]

**Table 2:** Animal models used to evaluate the effect of HEV infection on the male reproductive system.

Animal Model	Virus/Source	Findings	Sign of Infertility or Testes Damage	Reference
Pigs 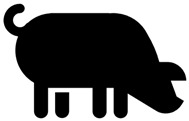	GT4, subtype 4i	○ Naturally infected. ○ HEV RNA in semen.	No	[63]
	GT3, US2 strain Human derived Gastrointestinal	○ Experimental Infection. ○ HEV RNA in sperm cells and seminal fluid, and HEV ORF2 in sperm head.	Yes	[64]
	GT3 Wild boar derived Liver homogenate	○ Experimental infection. ○ No HEV RNA in testis.	No	[52]
Mongolian gerbils ** 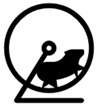 **	GT4 Swine-derived Liver homogenate	○ Experimental Infection. ○ HEV RNA in testes.	Yes	[73]
	GT3- subtype 3b human-derived fecal preparation	○ Experimental Infection. ○ HEV RNA in testes and epididymis	Not recorded	[50]
	GT3- subtype 3b human-derived supernatant of testis explants	○ Experimental Infection. ○ Supernatant of testis explants were infectious to gerbils	Not recorded	[50]
Non-human primates ** 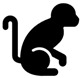 **	GT4 Swine-derived Fecal preparations	○ Experimental Infection. ○ HEV RNA in testes and epididymis	Yes	[54]
Rabbit 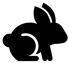	GT3- rabbit strain Fecal preparations	○ Experimental Infection. ○ HEV RNA in testes	Yes	[50]
Balb/c mice 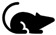	GT4 human-derived Fecal preparations	○ Experimental Infection. ○ HEV RNA in testes, epididymis, seminal vesicles, and epididymal fluid.	Yes	[81]

## Data Availability

No new data were generated in the manuscript. For any inquiries, please contact the corresponding authors.
